# Localization and Molecular Cloning of the *ASMT* Gene for Melatonin Synthesis in Pigs

**DOI:** 10.3390/ijms26020606

**Published:** 2025-01-13

**Authors:** Laiqing Yan, Guangdong Li, Shoulong Deng, Likai Wang, Yiwei Wang, Zixia Shen, Depeng Yin, Pengyun Ji, Bingyuan Wang, Guoshi Liu

**Affiliations:** 1 State Key Laboratory of Animal Biotech Breeding, Key Laboratory of Animal Genetics, Breeding and Reproduction of the Ministry of Agriculture, Frontiers Science Center for Molecular Design Breeding (MOE), College of Animal Science and Technology, China Agricultural University, Beijing 100193, China; laiqingyan@cau.edu.cn (L.Y.); 15600911225@cau.edu.cn (G.L.); 15771397288@163.com (L.W.); s20233040771@cau.edu.cn (Y.W.); shenzixiaa@163.com (Z.S.); yindepeng01@163.com (D.Y.); jipengyun@cau.edu.cn (P.J.); 2 National Center of Technology Innovation for Animal Model, National Health Commission of China (NHC) Key Laboratory of Comparative Medicine, Institute of Laboratory Animal Sciences, Chinese Academy of Medical Sciences and Comparative Medicine Center, Peking Union Medical College, Beijing 100021, China; dengshoulong@cnilas.org

**Keywords:** pig, melatonin, melatonin-synthesizing enzyme, *ASMT*

## Abstract

Melatonin is synthesized in multiple tissues and organs of pigs, and existing studies have shown the presence of the melatonin-synthesizing enzyme ASMT protein. However, the genomic information for the *ASMT* gene has been lacking. The aim of this study was to locate the genomic information of the *ASMT* gene in pigs using comparative genomics analysis and then obtain the coding region information through molecular cloning. First, using the NCBI Genome Data Viewer, we found that in most animals, the *AKAP17A* gene is often located next to the *ASMT* gene, with both genes arranged in the same direction. Similarly, the *P2RY8* gene is commonly adjacent to the *ASMTL* gene, also in the same orientation. We also discovered that the *ASMTL* gene is frequently adjacent to the ASMT gene and arranged in the opposite direction. Using the “three-point localization” principle, we inferred the position of the *ASMT* gene based on the coordinates of *AKAP17A* and *ASMTL* in pigs. Our results revealed that on the pig X chromosome, a gene called LOC110258194 is located next to the *AKAP17A* and *ASMTL* genes, and its arrangement aligns with the *ASMT* gene in other species. Additionally, Ensembl contains a gene, ENSSSCG00000032659, at the same position, with completely overlapping exons, though it is not annotated as *ASMT*. Further analysis using the TreeFam tool from EMBL-EBI and the CDD tool from NCBI revealed that LOC110258194 and ENSSSCG00000032659 do not contain the typical Maf domain of *ASMTL* and, thus, should not be annotated as *ASMTL*, but rather as the *ASMT* gene. Using a slow-down PCR method for high-GC content genes, we successfully cloned the full CDS region of the pig *ASMT* gene and identified a new transcript missing Exon 6 and Exon 7. This transcript was submitted to NCBI and assigned the GenBank accession number MW847601. Our results represent the first successful localization of the *ASMT* gene in pigs, the first cloning of the *ASMT* gene’s coding region, and the first discovery of a new transcript of the pig *ASMT* gene.

## 1. Introduction

Melatonin, also known as N-acetyl-5-methoxytryptamine, is an indoleamine hormone primarily secreted by the pineal gland and is widely found in nature. The synthesis of melatonin begins with tryptophan, which is converted into 5-hydroxytryptophan by the enzyme tryptophan hydroxylase. This compound is then converted into 5-hydroxytryptamine (5-HT), which is acetylated. The compound is then converted into N-acetyl-5-hydroxytryptamine (NAS) by arylamine N-acetyltransferase (AANAT), and finally transformed into melatonin by N-acetylserotonin O-methyltransferase (ASMT) [1]. AANAT is considered the rate-limiting enzyme in melatonin synthesis because its expression or activity in the pineal gland follows a circadian rhythm that matches the rhythm of serum melatonin in most tested animals. In mammals, other non-specific N-acetyltransferases, including arylamine N-acetyltransferases 1 and 2 (NAT1, NAT2), appear to also contribute to serotonin acetylation and melatonin synthesis [2]. Therefore, even in the absence of AANAT, animals can still synthesize considerable levels of melatonin. For example, in C57BL/6 mice, where AANAT is naturally truncated and catalytically inactive, melatonin can still be detected in their skin tissues and blood, albeit at lower levels [3,4]. ASMT, also known as hydroxyindole-O-methyltransferase (HIOMT), uses S-adenosylmethionine (SAM) as a cofactor and represents another rate-limiting step in melatonin synthesis. Evidence suggests that, in some cases, particularly in hamsters, ASMT may play a more critical role than AANAT in regulating melatonin production [5,6]. Currently, no other enzymes involved in the N-acetylserotonin O-methylation process used to form melatonin in mammals have been identified. This finding reinforces the argument that ASMT is the most efficient and specific rate-limiting enzyme in melatonin synthesis, particularly under conditions of high production rates [7]. Melatonin, as an antioxidant, plays a crucial role in the antioxidant processes of organisms. In addition to its antioxidant activity, melatonin has various other functions, including regulation of circadian rhythms, enhancement of immune function, regulation of reproduction, promotion of sleep, and anti-inflammatory effects. All these functions were acquired at different stages of evolution [8].

Melatonin has a wide range of applications in improving pig production performance, including enhancing reproductive performance, alleviating heat stress, reducing piglet diarrhea, improving gut microbiota, boosting immunity, and mitigating gamete and embryo cryodamage [9,10]. In previous studies, our team fed melatonin in the form of feed additives starting from the time of sow weaning until 10 days after mating. The results showed that feeding high doses of melatonin increased the average litter size by 1.16 piglets and the average litter weight by 1.3 kg [11]. Additionally, in vitro melatonin supplementation significantly improved the quality of porcine oocytes, promoted oocyte maturation, stimulated granulosa cells to produce progesterone, maintained luteal function, enhanced blastocyst quality, and improved early embryo development efficiency [12,13]. Therefore, studying the synthesis and mechanism of melatonin in pigs is crucial, and ASMT, as a key enzyme in the melatonin synthesis pathway, plays an irreplaceable role. However, despite the existing research showing that multiple organs and tissues in animals can synthesize and secrete melatonin [14,15,16], and the presence of the ASMT enzyme, genomic information for this gene is missing from databases, hindering related studies on melatonin synthesis in pigs. This study aims to clarify the genomic information of the pig *ASMT* gene, clone its coding region, and provide a theoretical reference for investigating melatonin synthesis in pigs and its potential to improve sow production.

## 2. Results

### 2.1. NCBI Database Search Fails to Identify Pig ASMT

**Figure 1:**
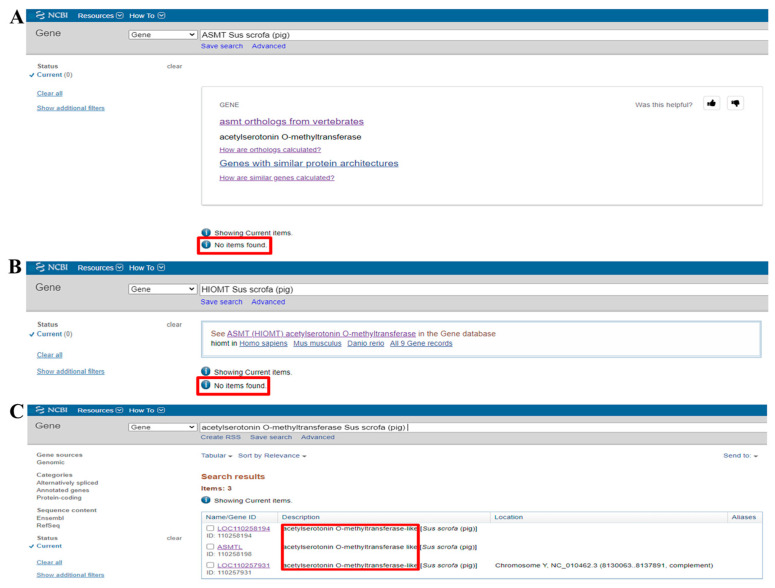
The search results for different forms of the pig ASMT gene in the NCBI database. (**A**) The search results for the pig ASMT abbreviation in the NCBI database. (**B**) The search results for another name for pig ASMT in the NCBI database. (**C**) The search results for the full name of the pig ASMT gene in the NCBI database.

Initially, we searched for the pig *ASMT* gene in the NCBI database. As shown in Figure 1A, the search returned “No items found”. We then searched using another name for ASMT, HIOMT, but again no results were found, as shown in Figure 1B. Finally, we searched for the full name of the *ASMT* gene, acetylserotonin O-methyltransferase, which led to the identification of the *ASMTL* (acetylserotonin O-methyltransferase-like) gene, as shown in Figure 1C.

### 2.2. Discrepancies in Pig ASMT Gene Information on Google Scholar

**Figure 2:**
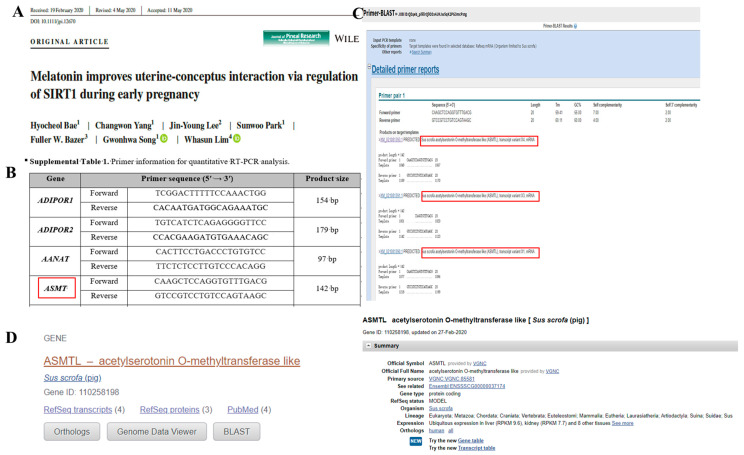
The literature search and primer sequence alignment for the pig ASMT gene. (**A**) The only paper found on Google Scholar related to pig ASMT gene expression research. (**B**) The quantitative primer sequence information for the ASMT gene from the paper. (**C**) The Primer-BLAST results obtained using the ASMT gene primer information from the paper, which were aligned to ASMTL. (**D**) Pig ASMTL gene information from the NCBI database.

A search through the literature on pig *ASMT* gene mRNA using Google Scholar returned only one relevant paper: Melatonin improves uterine-conceptus interaction via regulation of SIRT1 during early pregnancy [17]. Using the primer sequences for the pig ASMT gene provided in that paper (Figure 2A,B), we performed Primer-BLAST. The results, shown in Figure 2C,D, revealed that the aligned gene was *ASMTL*. Further searches for pig *ASMTL* identified it as an independent protein-coding gene (Gene ID: 110258198) with three transcripts: XM_021081390.1, XM_021081391.1, and XM_021081393.1. The genomic location is NW_018084901.1 (798964..820604, complement), indicating that the authors of the paper mistakenly identified pig *ASMTL* as the *ASMT* gene.

### 2.3. AKAP17A Gene Is Often Found near ASMT Gene

**Figure 3:**
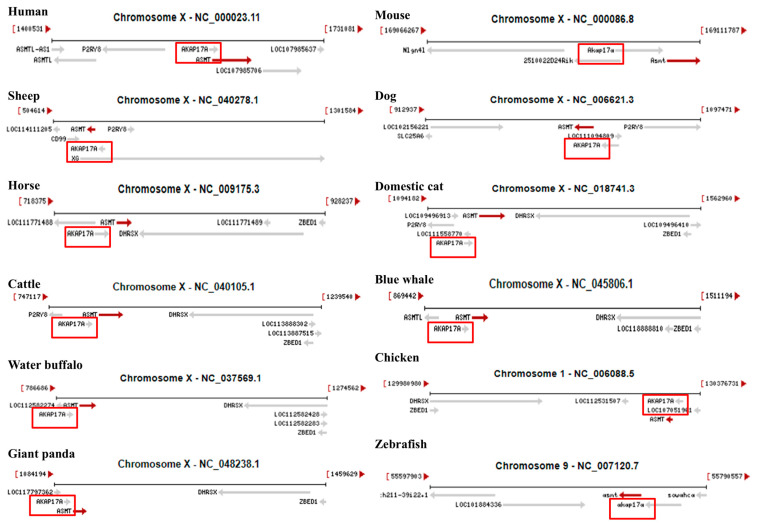
Vertical comparison of ASMT gene coordinates and chromosomal arrangement patterns across different species.

Using the NCBI Genome Data Viewer, we retrieved *ASMT* gene data from various species, including humans (*Homo sapiens*), sheep (*Ovis aries*), horses (*Equus caballus*), cattle (*Bos taurus*), buffalo (*Bubalus bubalis*), giant pandas (*Ailuropoda melanoleuca*), mice (*Mus musculus*), dogs (*Canis lupus familiaris*), domestic cats (*Felis catus*), blue whales (*Balaenoptera musculus*), chickens (*Gallus gallus*), and zebrafish (*Danio rerio*). We then analyzed and compared the chromosomal locations of the *ASMT* gene and its neighboring genes. As shown in Figure 3, the results revealed that in most mammals, including humans, sheep, horses, cattle, buffalo, giant pandas, mice, dogs, domestic cats, and blue whales, the *ASMT* gene is primarily located on the X chromosome. Furthermore, it was observed that the AKAP17A gene is often found adjacent to the *ASMT* gene, and the two genes are consistently oriented in the same direction, regardless of whether the species is mammalian, avian, or piscine.

### 2.4. Vertical Comparison of ASMT Gene Coordinates and Chromosomal Arrangement Patterns Across Different Species

**Figure 4:**
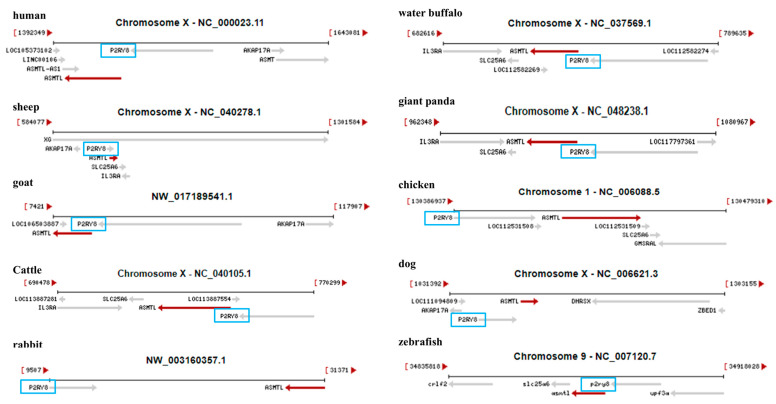
Vertical comparison of ASMTL gene coordinates and chromosomal arrangement patterns across different species.

Additionally, using the NCBI Genome Data Viewer, we retrieved *ASMTL* gene data from various species, including humans (*Homo sapiens*), sheep (*Ovis aries*), goats (*Capra hircus*), cattle (*Bos taurus*), rabbits (*Oryctolagus cuniculus*), buffalo (*Bubalus bubalis*), giant pandas (*Ailuropoda melanoleuca*), chickens (*Gallus gallus*), dogs (*Canis lupus familiaris*), and zebrafish (*Danio rerio*). We analyzed and compared the chromosomal locations of *ASMTL* and its neighboring genes. As shown in Figure 4, the results revealed that in most species, including humans, sheep, goats, cattle, rabbits, buffalo, giant pandas, chickens, dogs, and zebrafish, the *ASMTL* gene is primarily located on the X chromosome. Furthermore, it was observed that the P2RY8 gene is often found adjacent to the *ASMTL* gene, and the two genes are consistently oriented in the same direction, regardless of whether the species is mammalian, avian, or piscine.

### 2.5. ASMT and ASMTL Genes Are Often Adjacent and Arranged in Opposite Directions

**Figure 5:**
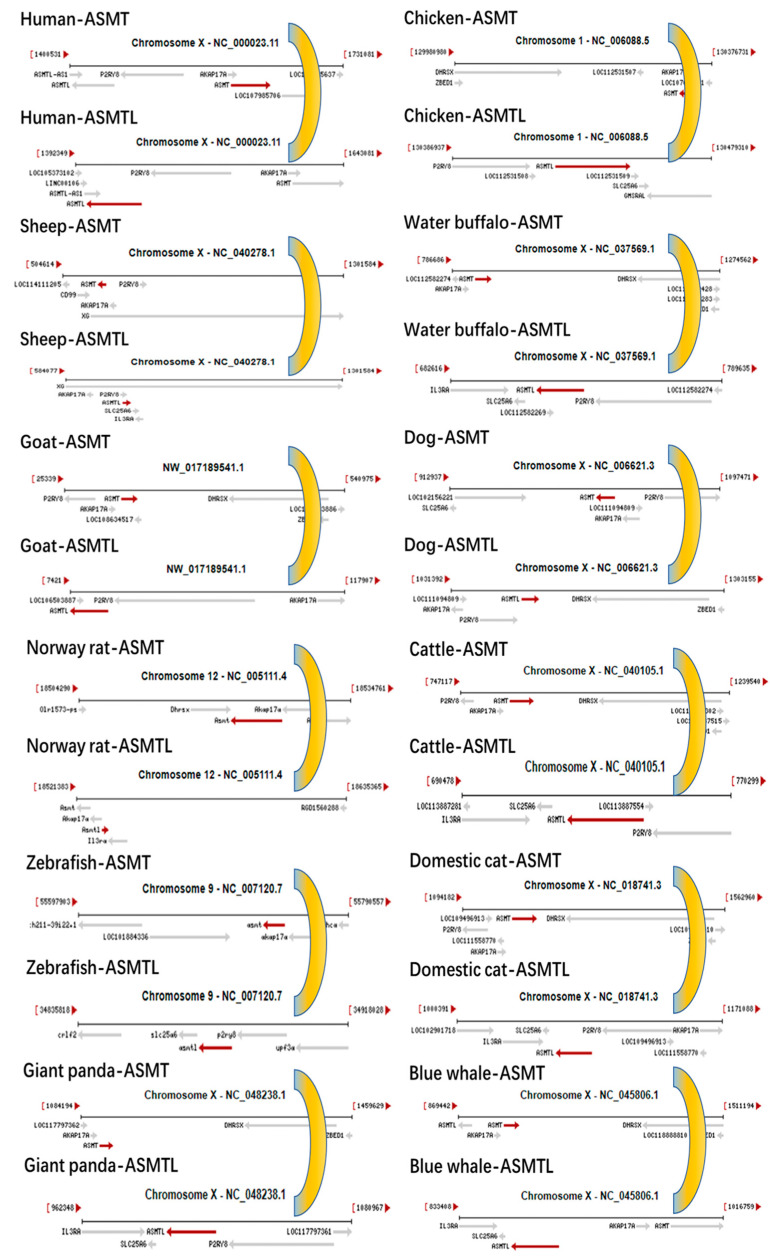
Horizontal and vertical comparison of the relative positions of the ASMT-ASMTL genes within the same species and across different species.

Interestingly, we observed that the *ASMT* and *ASMTL* genes in humans are located on the same chromosome and are adjacent to each other. We then further compared the chromosomal arrangement of the *ASMT* and *ASMTL* genes across various species, including sheep (*Ovis aries*), goats (*Capra hircus*), Norway rats (*Rattus norvegicus*), zebrafish (*Danio rerio*), giant pandas (*Ailuropoda melanoleuca*), chickens (*Gallus gallus*), buffalo (*Bubalus bubalis*), dogs (*Canis lupus familiaris*), cattle (*Bos taurus*), domestic cats (*Felis catus*), and blue whales (*Balaenoptera musculus*). As shown in Figure 5, the results revealed that in all these species, the *ASMT* and *ASMTL* genes are located on the same chromosome, are positioned closely together, and are arranged in opposite directions.

### 2.6. The Pig ASMT Gene Is Located in the PAR Region of the X Chromosome

**Figure 6:**
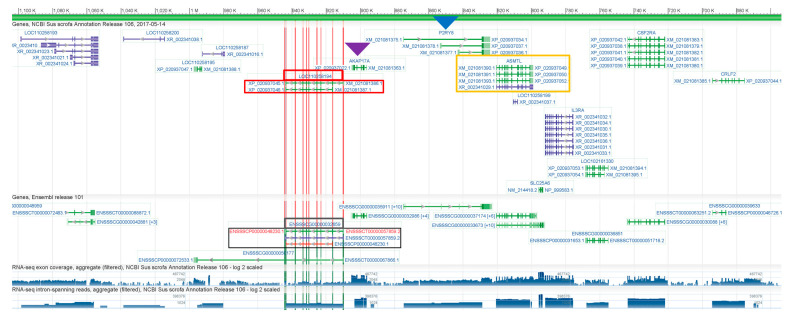
Localization of the pig ASMT gene.

Based on the observed pattern of *ASMT* and *ASMTL* genes being adjacent and arranged in opposite directions across different species, we hypothesized that pigs would follow a similar pattern. We then used the NCBI Graphical Sequence Viewer to retrieve the pig *ASMTL* gene, as shown in Figure 6. Based on the “three-point positioning” rule, where *AKAP17A* (indicated by the purple arrow) is often found near *ASMT*, and *P2RY8* (indicated by the blue arrow) is commonly associated with *ASMTL* (outlined in the orange box), we proceeded with our analysis. Following this “three-point positioning” rule, we discovered that on the pig X chromosome, near the *AKAP17A* and *ASMTL* genes, there is indeed a gene called LOC110258194 (highlighted by the red box). The positioning of this gene follows the same arrangement as the *ASMT* gene in other species. Furthermore, in the Ensembl database, a gene called ENSSSCG00000032659 (outlined in the black box) is located at the same position, and the exons of both genes are perfectly aligned (shown by the red vertical lines indicating exon positions). Our next step is to confirm that both NCBI LOC110258194 and Ensembl ENSSSCG00000032659 are indeed the pig *ASMT* gene.

**Figure 7:**
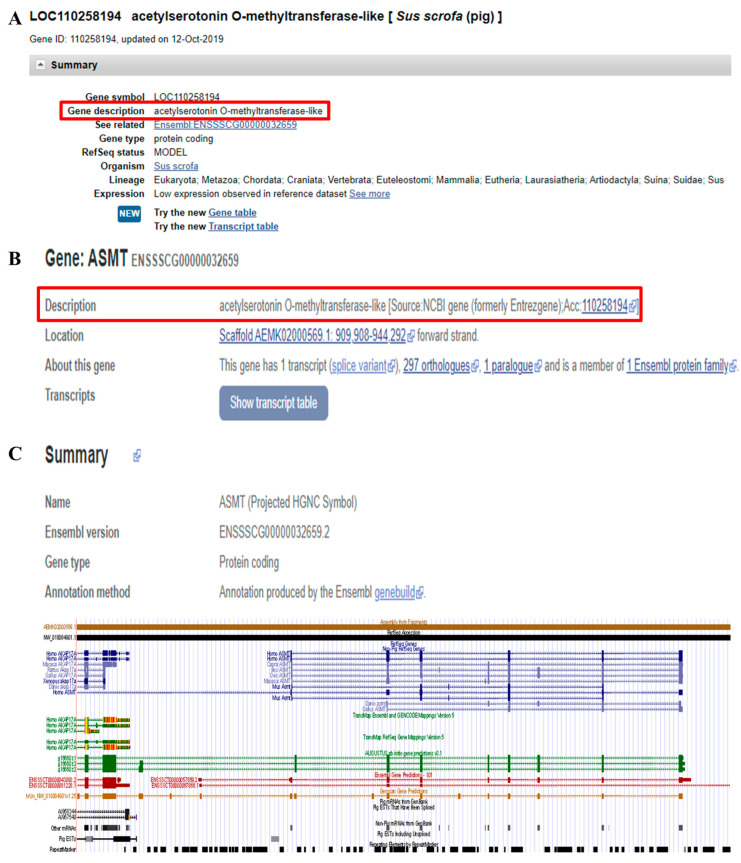
NCBI LOC110258194, Ensembl ENSSSCG00000032659, and UCSC search results. (**A**) The NCBI LOC110258194 gene description is ASMTL. (**B**) The Ensembl ENSSSCG00000032659 gene description is ASMTL. (**C**) The UCSC Genome Browser shows that the ASMT gene is at the same genomic location across different species (human, goat, cattle, sheep, rhesus macaque, mouse).

We searched for pig LOC110258194 in the NCBI database and found that its gene description is not *ASMT* (acetylserotonin O-methyltransferase), but rather *ASMTL* (acetylserotonin O-methyltransferase-like) (Figure 7A). Furthermore, although the gene name of ENSSSCG00000032659 in the Ensembl database is *ASMT*, its gene description is still listed as *ASMTL* (Figure 7B). Since the pig *ASMTL* gene is already independently annotated, it is clear that both the NCBI and Ensembl databases have mistakenly annotated the pig *ASMT* gene as *ASMTL*. We further validated this using the UCSC Genome Browser, which confirmed that the *ASMT* gene is indeed at the same genomic location across different species (Figure 7C).

**Figure 8:**
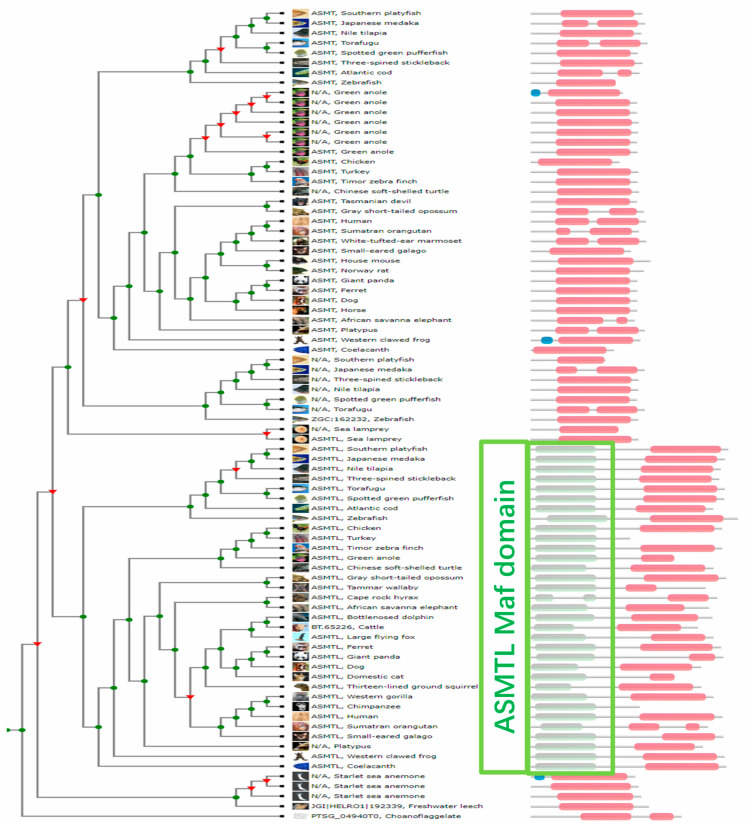
Evolutionary tree and protein domains of ASMT and ASMTL genes across 42 different species.

**Figure 9:**
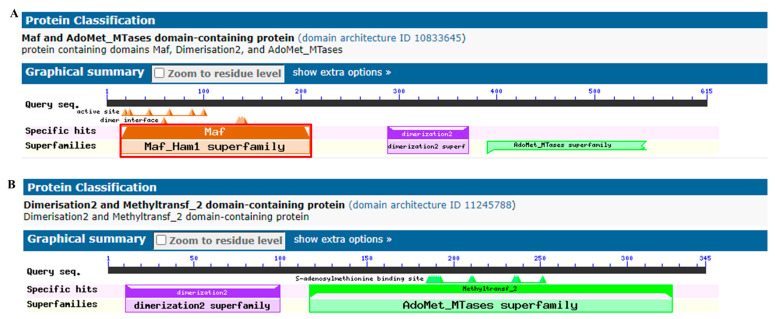
CDD analysis of pig ASMTL (**A**) and LOC110258194 (**B**).

To further confirm that both the NCBI and Ensembl databases mistakenly annotated the pig *ASMT* gene as *ASMTL*, we distinguished these two genes based on their protein domains. We used the EMBL-EBI TreeFam tool (https://www.treefam.org/) to construct an evolutionary tree and perform protein domain analysis for the *ASMT* and *ASMTL* genes of 42 different species, as shown in Figure 8. The results showed that the main domain of *ASMT* in different species is Methyltransf_2 (highlighted by a red stripe in the figure), while *ASMTL* has an additional unique conserved domain, Maf (highlighted by a light green stripe). Similarly, we performed a CDD analysis (https://www.ncbi.nlm.nih.gov/Structure/cdd/cdd.shtml, accessed on 5 December 2023) on pig *ASMTL* and LOC110258194 The analysis revealed that the pig ASMTL protein contains the Maf domain (Figure 9A), whereas the protein encoded by the LOC110258194 gene does not have the Maf domain (Figure 9B). This further confirmed the error in the machine annotation by the NCBI database, indicating that LOC110258194 should be annotated as *ASMT*, not *ASMTL*. It is located on the pig X chromosome at the coordinates NW_018084901.1 (909502..943966).

### 2.7. Molecular Cloning of the Pig ASMT Gene

**Figure 10:**
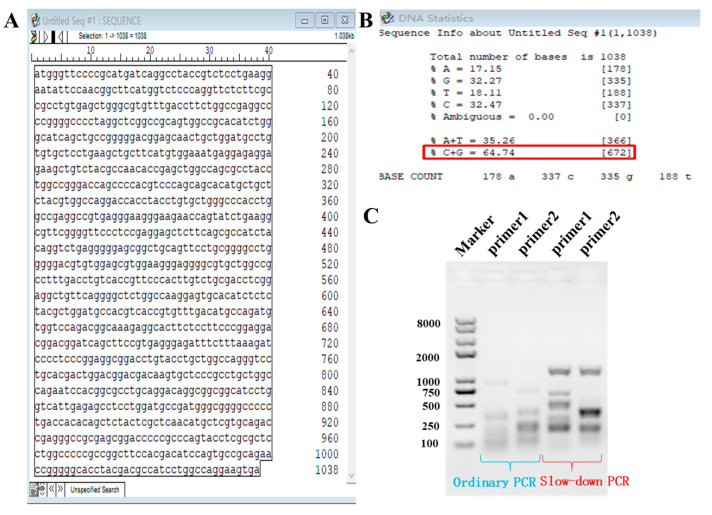
Sequence analysis and molecular cloning of the pig ASMT gene. (**A**) The CDS region sequence information of the pig ASMT gene from NCBI. (**B**) The GC content analysis of the CDS region of the ASMT gene. (**C**) The molecular cloning results of the ASMT gene.

**Figure 11:**
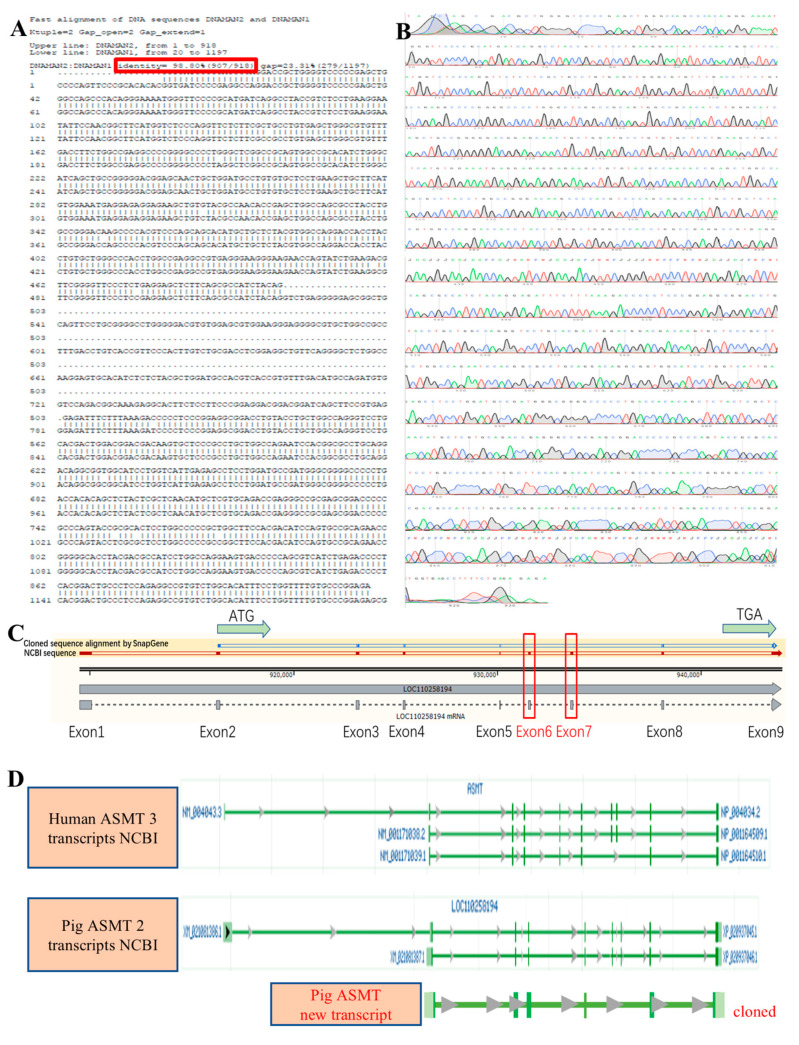
Sequencing results and alignment analysis of the pig ASMT gene cloning. (**A**) The alignment of the cloned pig ASMT gene sequence with the reference sequence. (**B**) The Sanger sequencing chromatogram of the cloned pig ASMT gene sequence. (**C**) Visualization of the exon information of the cloned pig ASMT gene using SnapGene software. (**D**) Comparison of human ASMT gene transcripts and the existing and newly cloned pig ASMT transcripts in NCBI.

Previous research has confirmed that pig LOC110258194 is the *ASMT* gene (CDS sequence information shown in Figure 10A). The next step was to perform molecular cloning. We collected pig ovarian tissue, extracted total RNA, and obtained the cDNA amplification template. Initially, we designed two pairs of primers spanning the CDS region, but regular PCR amplification failed for both (Figure 10C, lanes 1 and 2). We analyzed the structure of the pig LOC110258194 (*ASMT*) gene using Editseq (DNASTTAR V7.1) software and found that, similar to the mouse *ASMT* gene, the pig *ASMT* gene has a high GC content (64.74%, Figure 10B). Therefore, we used the previously developed slow-down PCR method (patented) for cloning high-GC content genes, and both primer pairs successfully amplified the gene (Figure 10C, lanes 3 and 4). After cloning the sequence, we performed Sanger sequencing (chromatogram shown in Figure 11B) and aligned the results with the target sequence. Using DNAman 9.0 software, the alignment resulted in a 98.80% match (Figure 11A). We noticed a missing segment in the sequence and performed visual alignment using SnapGene 4.1.9 software. The results showed that the cloned sequence was missing Exons 3 and 4 from the end (Figure 11C), suggesting that this is a new transcript. As shown in Figure 11D, an analysis of the three human ASMT transcript variants in the NCBI database revealed that pigs already have two transcripts. The newly discovered transcript corresponds exactly to the third human transcript (which also misses Exons 3 and 4 at the end). This finding further confirms the authenticity of the new transcript.

## 3. Discussion

In addition to providing meat and other products for humans, pigs are also an essential animal model in biomedical and pharmaceutical research [18]. The feeding and the management of sows during the reproductive period are crucial to improving sow reproductive capacity. Recent studies have highlighted the crucial role of melatonin in enhancing pig fertility, making it essential to explore its synthesis and function within pigs to improve reproductive outcomes. In addition, melatonin also plays an important role in improving human reproductive diseases [19]. The classic biosynthesis pathway of melatonin in mammals involves four key steps, with the final enzyme, ASMT (acetylserotonin O-methyltransferase), playing a critical role. The *ASMT* gene is located on the sex chromosomes, particularly on the X and Y chromosomes. Specifically, it resides in pseudoautosomal region 1 (*PAR1*), which is present on both sex chromosomes [20]. Additionally, a homologous sequence has been detected in the subtelomeric region of mouse chromosome 9, with weak binding to the corresponding fluorescence in situ hybridization (FISH) probe [21]. It remains unclear whether this sequence has functional activity. To date, no isoenzymes of *ASMT* have been identified in mammals, though *ASMT* isoforms have been identified in fish (*ASMT1* and *ASMT2*) [22]. This further underscores the critical role of *ASMT* in the melatonin biosynthesis pathway. In other animals, such as mice, rats, rhesus monkeys, cattle, sheep, goats, chickens, ducks, and fish, the genes encoding enzymes involved in melatonin synthesis have been successfully identified and cloned. The mouse Asmt gene is located in the pseudoautosomal region (PAR) of the X chromosome, an area characterized by high recombination and mutation rates [23]. This region remains enigmatic, and as a result, the mouse Asmt gene was not successfully mapped and cloned until 2010 [24]. For pigs, which have significant value both as a food source and in medicine, the *ASMT* gene has yet to be identified in the NCBI database. We hypothesize that the pig *ASMT* gene may be similar to the mouse *ASMT* gene, and the high mutation rate in this complex region could be the reason it has not been successfully discovered. This has contributed to the stagnation of research into the melatonin biosynthesis pathway in pigs.

The implementation of the Human Genome Project marked the beginning of the genomic era [25]. With the continuous advancement of sequencing technologies and the reduction in sequencing costs, the genomes of an increasing number of species are being sequenced. The generation of genomic data from prokaryotes, eukaryotes, and other diverse species has broadened our understanding of species evolution and the origins of life [26], thereby advancing comparative genomics research. Comparative genomics refers to the comparative analysis of structural and functional gene regions across the genomes of multiple individuals (populations) of a single species or across the genomes of multiple species, based on genomic maps and sequencing technologies [27]. These structural and functional gene regions include DNA sequences, genes, gene families, gene orders, regulatory sequences, and other genomic structural markers. Comparative genomics analysis, specifically, uses bioinformatics methods to compare the structural features of genomes from multiple species, identifying similarities and differences. This allows the study of gene families’ contraction and expansion, divergence times, evolutionary relationships, and the generation and evolution of new genes [28,29]. Degenerate primers are a primer design approach used for cloning unknown genes [30], based on the redundancy of the genetic code. In some cases, primers are designed by reverse engineering DNA sequences from conserved amino acid sequences. Since most amino acids are encoded by more than one codon, reverse engineering DNA sequences from amino acid sequences results in ambiguity in some base positions. Therefore, the primers designed in this way are actually mixtures of multiple sequences. Earlier, we attempted to clone the pig ASMT gene by designing degenerate primers based on conserved ASMT amino acid sequences from multiple species, but these attempts were unsuccessful. Later, we shifted our approach, using comparative genomics analysis to predict the localization pattern of the pig *ASMT* gene, which was then verified through molecular cloning. This ultimately allowed us to determine the gene’s location and sequence information in the pig genome. Interestingly, there are three transcripts of the human *ASMT* gene: transcript 1 (ten exons), transcript 2 (nine exons), and transcript 3 (seven exons) [30]. To date, there are two known transcripts of the pig ASMT gene: transcript 1 (nine exons) and transcript 2 (eight exons). Based on comparative genomics principles, we hypothesized that if human *ASMT* exhibits strong conservation, the pig *ASMT* gene should also have a third transcript (transcript 3) with six exons. Our molecular cloning results identified a new transcript missing the third and fourth exons, with exactly six exons, which confirms our hypothesis and further validates the existence of this new transcript. In summary, we resolved the misannotation of the ASMT gene in the database, cloned the full CDS region of the pig *ASMT* gene, and identified a new transcript missing Exon 6 and Exon 7. This was submitted to NCBI and assigned the GenBank accession number MW847601.

## 4. Materials and Methods

### 4.1. Preparation of Common Reagents and Solutions

Strain: DH5α; plasmid: T-vector; standard Taq polymerase: Kangwei Century Co., Ltd., Beijing, China; high-fidelity Taq polymerase: TaKaRa Co., Ltd., Tokyo, Japan; KOD polymerase: KOD, Aichi, Japan; molecular weight markers: 1 Kb Ladder, DS15000 (Dongsheng Guangzhou Dongsheng Biotechnology Co., Ltd., Guangzhou, China), Trans2K Plus (Fulinggold Co., Hongkong, China). Ethidium bromide (EB): prepare a stock solution of 10 mg/mL using deionized water, then dilute to 0.5 µg/mL for use. Electrophoresis buffer (50× TAE): dissolve 242.0 g Tris-Cl, 57.1 mL glacial acetic acid (A.P.), and 100 mL of 0.5 M EDTA (pH 8.0) in water, then adjust the volume to 1 L. 1.2% agarose gel: weigh 0.6 g of agarose powder (BIOWEST, Madrid, Spain) into a 100 mL flask, add 50 mL of 1× TAE buffer, heat in a microwave until the solid particles are completely dissolved, then cool to about 60 °C before casting the gel. DNA fragment recovery kit: OMEGA Bio-tek, Norcross, GA, USA; plasmid DNA mini-prep kit: Tiangen Biotech, Beijing, China. Ampicillin solution (AMP): prepare a 100 mg/mL solution in sterilized water, aliquot, and store at −20 °C. LB medium: Dissolve 10.0 g of peptone, 5.0 g of yeast extract, and 10.0 g of NaCl in 800 mL of water. For solid medium, add 15.0 g of agar powder. Adjust the pH to 7.5, then adjust the volume to 1 L and autoclave. 80% glycerol (*w*/*v*): mix 80 mL of glycerol with 20 mL of water, thoroughly mix, and autoclave. M-MLV reverse transcriptase, RNase inhibitor: Promega Corp., Madison, WI, USA; DEPC: Amresco Inc., Cincinnati, OH, USA. All other chemical reagents, unless otherwise specified, were purchased from Sigma-Aldrich, Burlington, MA, USA.

### 4.2. Software and Websites for Experimental Analysis

(1) Gene Query and Analysis Websites:
  NCBI (https://ncbi.nlm.nih.gov/);
  Ensembl (https://asia.ensembl.org/);
  UCSC Genome Browser (http://genome.ucsc.edu/).
(2) Primer Design Website: Primer 3.0 (https://primer3.ut.ee).
(3) Vector Design and Sequence Alignment Software: SnapGene.

### 4.3. Analysis of ASMT Gene Arrangement Across Different Species

A comparative analysis was conducted to examine the arrangement of *ASMT* genes across different species’ chromosomes. The *ASMT* genes from various species, including humans (*Homo sapiens*), sheep (*Ovis aries*), horses (*Equus caballus*), cattle (*Bos taurus*), water buffalo (*Bubalus bubalis*), giant pandas (*Ailuropoda melanoleuca*), mice (*Mus musculus*), dogs (*Canis lupus familiaris*), domestic cats (*Felis catus*), blue whales (*Balaenoptera musculus*), chickens (*Gallus gallus*), and zebrafish (*Danio rerio*), were retrieved from the NCBI Genome Data Viewer. The chromosomal locations of these genes, as well as the genes located in their proximity, were analyzed and compared.

### 4.4. Analysis of ASMTL Gene Arrangement Across Different Species

A comparative analysis was conducted to examine the arrangement of *ASMTL* genes across different species’ chromosomes. The *ASMTL* genes from various species, including humans (*Homo sapiens*), sheep (*Ovis aries*), goats (*Capra hircus*), cattle (*Bos taurus*), rabbits (*Oryctolagus cuniculus*), pigs (*Sus scrofa*), water buffalo (*Bubalus bubalis*), giant pandas (*Ailuropoda melanoleuca*), chickens (*Gallus gallus*), dogs (*Canis lupus familiaris*), and zebrafish (*Danio rerio*), were retrieved from the NCBI Genome Data Viewer. The chromosomal locations of these genes, as well as the genes in their vicinity, were analyzed and compared. Based on these findings, the likely location of the *ASMT* gene in pigs was inferred.

### 4.5. Phylogenetic Tree and Protein Domain Analysis

A phylogenetic tree for the ASMT and ASMTL genes of 42 different species, along with their protein domains, was constructed using the TreeFam tool from EMBL-EBI (https://www.treefam.org/). The conserved protein domains encoded by different genes were analyzed using the CDD tool from NCBI (https://www.ncbi.nlm.nih.gov/Structure/cdd/cdd.shtml).

### 4.6. RNA Extraction from Pig Ovaries

Ovarian tissue from pigs was collected from a slaughterhouse and homogenized. Approximately 50–100 mg of tissue was ground in liquid nitrogen, and 1 mL of TRIzol was added to lyse the cells. After lysis, 0.2 mL of chloroform was added to each 1 mL of TRIzol reagent. The sample tube was sealed and vigorously shaken by hand for 15 s, followed by a 15 min incubation period at room temperature. The sample was then centrifuged at 4 °C, 12,000× *g*, for 15 min. The supernatant was carefully transferred to a pre-prepared 1.5 mL RNase-free centrifuge tube, avoiding the lower phase to prevent contamination with impurities. Next, 0.5 mL of isopropanol (equal volume) was added, and the mixture was gently inverted and incubated at room temperature for 10 min. The sample was then centrifuged at 4 °C, 12,000× *g*, for 10 min. The supernatant was discarded and 1 mL of pre-cooled 75% ethanol prepared with RNase-free water was added. The sample was gently inverted 1–2 times to wash the pellet, then centrifuged at 4 °C, 7500× *g*, for 5 min. The remaining supernatant was carefully discarded, and the RNA pellet was air-dried. The pellet was then resuspended in RNase-free water by gently pipetting. RNA concentration and quality were measured using a NanoDrop spectrophotometer(Hangzhou Allsheng Instruments Co., Ltd., Hangzhou, China), and the integrity of the RNA samples was assessed by conventional agarose gel electrophoresis.

### 4.7. Reverse Transcription of RNA

(1) Removal of Genomic DNA from RNA

**Table:** 

Components	Volume (µL)
5× gDNA Eraser	2
gDNA Eraser Buffer	1
DEPC-Treated Water	1
RNA Template	6

Place the above reaction mixture into a PCR machine and incubate at 42 °C for 2 min.

(2) Reverse Transcription system

**Table:** 

Components	Volume (µL)
5 × PrimeScript^®^ Buffer 2	4
PrimeScript^®^ RT Enzyme Mix I	1
RT Primer Mix	1
DEPC-Treated Water	4
The reaction mixture from the previous step	10

Incubate the reaction mixture at 37 °C for 20 min and at 85 °C for 5 s in the PCR machine to obtain cDNA. Store at −20 °C.

### 4.8. Primer Design

(1) Details of primers

**Table:** 

Gene	Primer Sequence (5′–3′)	Accession. No	Product Length (bp)
ASMT	F1:CCCCAGTTCCCGCACAC	XM_021081386.1	1214
	R1:CAGAAGCTCAGCATCGCTCT		
	F2:CCCCAGTTCCCGCACAC		1351
	R2:GACCCCTCACTTCATCACATGCAA		

### 4.9. PCR Reaction System

(1) Specific response system

**Table:** 

Reagents	Volume
High-Fidelity Taq	25 μL
Primer-F	2 μL
Primer-R	2 μL
Template	2 μL
ddH_2_O	19 μL

(2) Specific response system

The PCR reaction program is as follows:

Pre-denaturation at 98 °C for 2 min.

98 °C 10 s, 72 °C 30 s, 72 °C 45 s, 4 cycles;

98 °C 10 s, 71 °C 30 s, 72 °C 45 s, 4 cycles;

98 °C 10 s, 70 °C 30 s, 72 °C 45 s, 4 cycles;

98 °C 10 s, 69 °C 30 s, 72 °C 45 s, 4 cycles;

98 °C 10 s, 68 °C 30 s, 72 °C 45 s, 4 cycles;

98 °C 10 s, 67 °C 30 s, 72 °C 45 s, 4 cycles;

98 °C 10 s, 66 °C 30 s, 72 °C 45 s, 4 cycles;

98 °C 10 s, 65 °C 30 s, 72 °C 45 s, 4 cycles.

After the cycles are complete, incubate at 72 °C for 10 min.

The PCR products are analyzed by electrophoresis on a 1.2% agarose gel. The target band is excised, purified, ligated into the T-vector, and sent for sequencing.

## 5. Conclusions

This study is the first to map the genomic location of the pig *ASMT* gene, clone its coding region, and discover a new transcript of the pig *ASMT* gene.

## Data Availability

The raw data supporting the conclusions of this article will be made available by the authors, without undue reservation.
